# Retraction: Pt NPs@GO as a highly efficient and reusable catalyst for one-pot synthesis of acridinedione derivatives

**DOI:** 10.1039/d1ra90148f

**Published:** 2021-10-07

**Authors:** Muharrem Kaya, Burak Aday

**Affiliations:** Biochemistry Department, Faculty of Arts and Science, Dumlupınar University Evliya Çelebi Campus 43100 Kütahya Turkey; Chemistry Department, Faculty of Arts and Science, Dumlupınar University Evliya Çelebi Campus 43100 Kütahya Turkey

## Abstract

Retraction for ‘Pt NPs@GO as a highly efficient and reusable catalyst for one-pot synthesis of acridinedione derivatives’ by Handan Pamuk *et al.*, *RSC Adv.*, 2015, **5**, 49295–49300, DOI: 10.1039/C5RA06441D.

Muharrem Kaya and Burak Aday hereby wholly retract this *RSC Advances* article due to concerns with the reliability of the data in the published article.

The high-resolution transmission electron micrograph inset in Fig. 1 that represents Pt NPs@GO is the same as Fig. 1b in a *Scientific Reports* article by Yasar Karatas, Hilal Acidereli, Mehmet Gulcan and Fatih Sen,^1^ which is a high-resolution transmission electron micrograph representing a PtRu@VC nanocatalyst. Fatih Sen provided replacement data for consideration. However, an expert reviewed the author’s response and concluded that it did not satisfactorily address the concerns, and that the replacement figure did not fully support the conclusions. Given the significance of the concerns about the validity of the data, the findings presented in this paper are no longer reliable.

Muharrem Kaya approves the retraction of this article due to concerns with the structural characterisation of the catalyst and therefore the conclusions presented may not be valid. Muharrem Kaya also states that the figures under question are out of their expertise and that they did not contribute to the data collection or processing of these figures. Muharrem Kaya contributed to the evaluation of the catalytic performances of the composite materials only.

Burak Aday states that the figures under question are out of their expertise and they did not contribute to the data collection and processing of Fig. 1. Burak Aday contributed to the evaluation of the catalytic performances of the composite materials. Burak Aday also states that since the structural characterisation of the catalyst is under question the conclusions derived in the manuscripts may not be valid.

Fatih Sen opposes this retraction. Handan Pamuk was contacted but did not respond.

Signed: Muharrem Kaya and Burak Aday

Date: 23^rd^ September 2021

Retraction endorsed by Laura Fisher, Executive Editor, *RSC Advances*

## Supplementary Material

## References

[cit1] Karatas Y., Acidereli H., Gulcan M., Sen F. (2020). Sci. Rep..

